# Re‐engineering a transferase scaffold for indole C3 methylation in diketopiperazines

**DOI:** 10.1002/pro.70254

**Published:** 2025-08-28

**Authors:** Mona Haase, Oliver H. Weiergräber, Jörg Pietruszka

**Affiliations:** ^1^ Institute of Bioorganic Chemistry & Bioeconomy Science Center (BioSC) Heinrich Heine University Düsseldorf in Forschungszentrum Jülich Jülich Germany; ^2^ Institute of Biological Information Processing (IBI‐7: Structural Biochemistry), Forschungszentrum Jülich Jülich Germany; ^3^ Institute of Bio‐ and Geosciences (IBG‐1: Bioorganic Chemistry), Forschungszentrum Jülich Jülich Germany

**Keywords:** biocatalysis, crystal structure, methyltransferase, pyrroloindole natural product, rational design

## Abstract

The pyrroloindole (hexahydropyrrolo[2,3‐*b*]indole, HPI) structural motif is present in a wide range of natural products with various biological activities, yet its chemical synthesis poses a challenge, particularly regarding methylation at the indole C3 position. In nature, S‐adenosyl methionine (SAM)‐dependent methyltransferases efficiently catalyze this reaction with high stereoselectivity. This study presents the investigation and rational re‐design of a potential methyltransferase, termed SeMT, from the actinomycete *Saccharopolyspora erythraea*. While its three‐dimensional structure elucidated via X‐ray crystallography confirmed extensive structural similarity to cyclic dipeptide‐processing methyltransferases such as SgMT, its putative catalytic center is clearly divergent. Accordingly, wild‐type SeMT displayed minimal activity with diketopiperazine (DKP) substrates, triggering an extensive mutagenesis effort aimed at iteratively enhancing this methyltransferase function. This work yielded a variant with appreciable activity, which was comprehensively characterized. Notably, a specific mutation within the catalytic triad of SeMT proved critical not only for its own function but also for the temperature‐activity profile of its homolog protein SgMT. Beyond the specific properties of SeMT, these findings hence provide important insights into the active center architecture of indole C3‐methyltransferases, supporting further development of these enzymes into refined biocatalysts for synthetic applications.

## 1 INTRODUCTION

**FIGURE 1:**
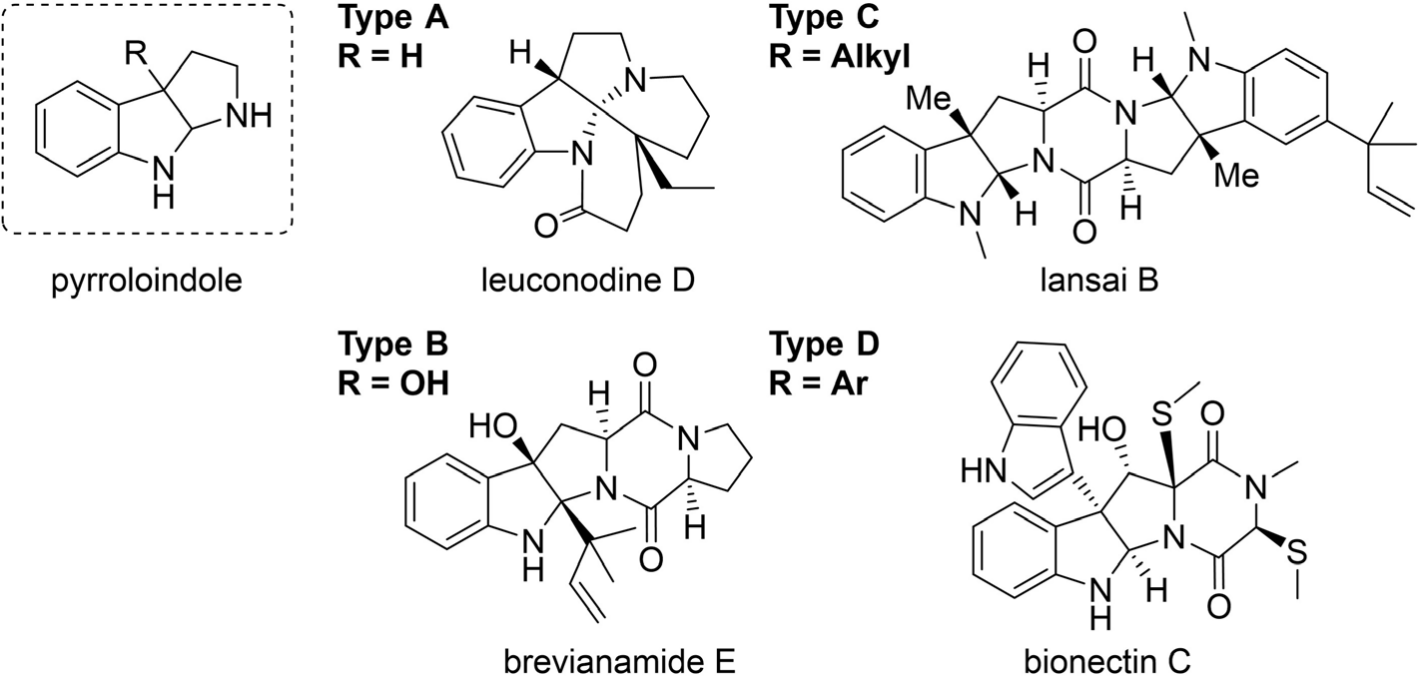
Selected natural products containing the pyrroloindole structural motif, categorized by their substituent at the indole C3 position.

The HPI structural motif is common in biologically active natural products, with its rigid structure enhancing affinity for various protein targets through hydrophobic interactions (Borthwick 2012; Mei et al. 2021; Ruiz‐Sanchis et al. 2011). HPI natural products can be classified based on the substituent at the C3 position (Figure 1): unsubstituted (R = H; type A), hydroxylated (R = OH; type B), alkylated (R = Alkyl; type C), arylated (R = Ar; type D), and dimeric pyrroloindoles (type E, not shown) (Amariei et al. 2024; Sun et al. 2022). The intriguing tricyclic structure and diverse biological activities of HPI compounds have made them attractive targets for synthesis, driving significant research efforts in the synthetic chemistry community (Mei et al. 2021). Biochemically, various enzyme classes—including cytochromes P450 (Fujimori et al. 2007; Guo et al. 2018), monooxygenases, methyltransferases (Amariei et al. 2022; Haase et al. 2023), and prenyltransferases (Liu et al. 2021; Yin et al. 2009)—play a role in the biosynthesis of these natural products by catalyzing the intramolecular cyclization of a substituted indole, leading to dearomatization and formation of the HPI motif (Sun et al. 2022). The stereoselective methylation at the indole C3 position remains a challenge with conventional organic chemistry methods, making C3‐methyltransferases a focal point of research in recent years (Amariei et al. 2022; Haase et al. 2023; Schatton et al. 2025). The methyltransferases SgMT from *Streptomyces griseoviridis* and StspM1 from *Streptomyces* sp. HPH0547, which catalyze C3‐methylation on tryptophan‐derived diketopiperazines, have been characterized and studied with regard to their mechanisms. Both enzymes exhibit similar structural features, as well as comparable pH and temperature profiles (Haase et al. 2023; Li et al. 2019).

In this study, the putative methyltransferase SeMT, a homolog of SgMT and StspM1, from *Saccharopolyspora erythraea* is investigated. Following the determination of its three‐dimensional structure, we explore the amenability of the SeMT scaffold to re‐engineering* indole C3‐methyltransferase activity by evaluating the impact of selected amino acid exchanges on its catalytic properties. The critical role of a conservative modification in the enzyme's catalytic triad is rationalized considering the unique structure of the SeMT N‐terminal segment, suggesting previously unanticipated ways of tuning the temperature profile of methyl transfer reactions.

## 2 RESULTS

### 2.1 Identification of a novel putative methyltransferase

A sequence‐based homology search was conducted on the enzymes SgMT and StspM1, which are indole C3‐methyltransferases known to accept a diketopiperazine substrate formed of two tryptophan residues. A putative methyltransferase from *Saccharopolyspora erythraea* NRRL 2338, henceforth termed SeMT, was identified featuring 56% sequence identity with both StspM1 and SgMT. Unlike these two methyltransferases, the gene encoding SeMT is not located within a typical gene cluster including a cyclic dipeptide synthase, prenyltransferase, and additional methyltransferase. The neighboring genes of SeMT encode an oxygenase of the MpaB family, a protein‐tyrosine phosphatase family protein, and transcriptional regulators; however, they do not indicate association with any known natural product. This suggests that SeMT might participate in a different, yet unknown biosynthetic pathway.

**FIGURE 2:**
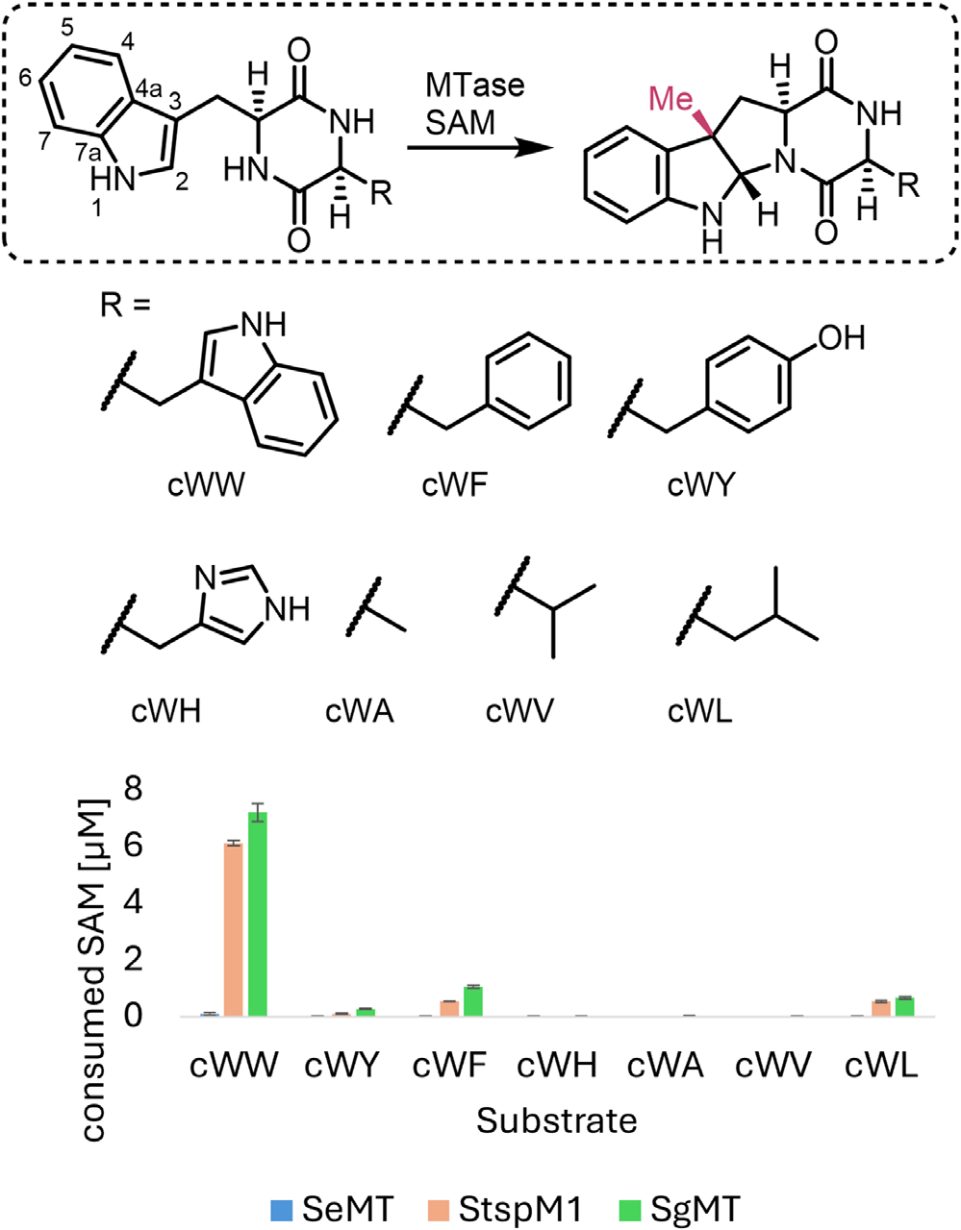
Methylation activity of SgMT, StspM1, and SeMT with seven different DKP substrates, denoted as cyclic (c) dipeptides. For the cyclization mechanism, see Data S1 (Figure S4). The activity was measured with the MTase Glo Assay (Promega), which detects the consumption of SAM. The reaction was carried out at 45°C for 15 min with an enzyme concentration of 3 μM.

To investigate its function, SeMT was heterologously expressed in *Escherichia coli*, purified, and tested for activity with seven different diketopiperazine substrates, as the natural substrate of SeMT could not yet be identified (Figures 2 and S2 as well as Table S4, Data S1). Substrate conversion rates were assessed using a commercially available bioluminescence‐based assay (MTase‐Glo Methyltransferase Assay; see section 5) throughout this work, unless noted otherwise. This assay detects the formation of S‐adenosyl homocysteine (SAH)—and hence the consumption of SAM—in the methyltransferase reaction. As reaction temperature, the optimal value established previously for both SgMT and StspM1 (45°C) was chosen; the effect of temperature on catalytic activity is discussed later in the manuscript. Intriguingly, SeMT did not show appreciable conversion for any of the substrates tested. The best‐accepted substrate is ll‐cycloditryptophan (cWW), still with 76 times less conversion than found for the homolog SgMT (Figure 2). Presuming that SeMT per se is a capable catalyst, our observations support the conjecture that indole‐substituted diketopiperazines are not among its genuine substrates.

To gain insight into the structural features underlying the disparate substrate scopes and enzymatic activities found for these enzymes, SeMT was crystallized, and its structure was determined via X‐ray crystallography.

### 2.2 X‐ray structure of SeMT

**FIGURE 3:**
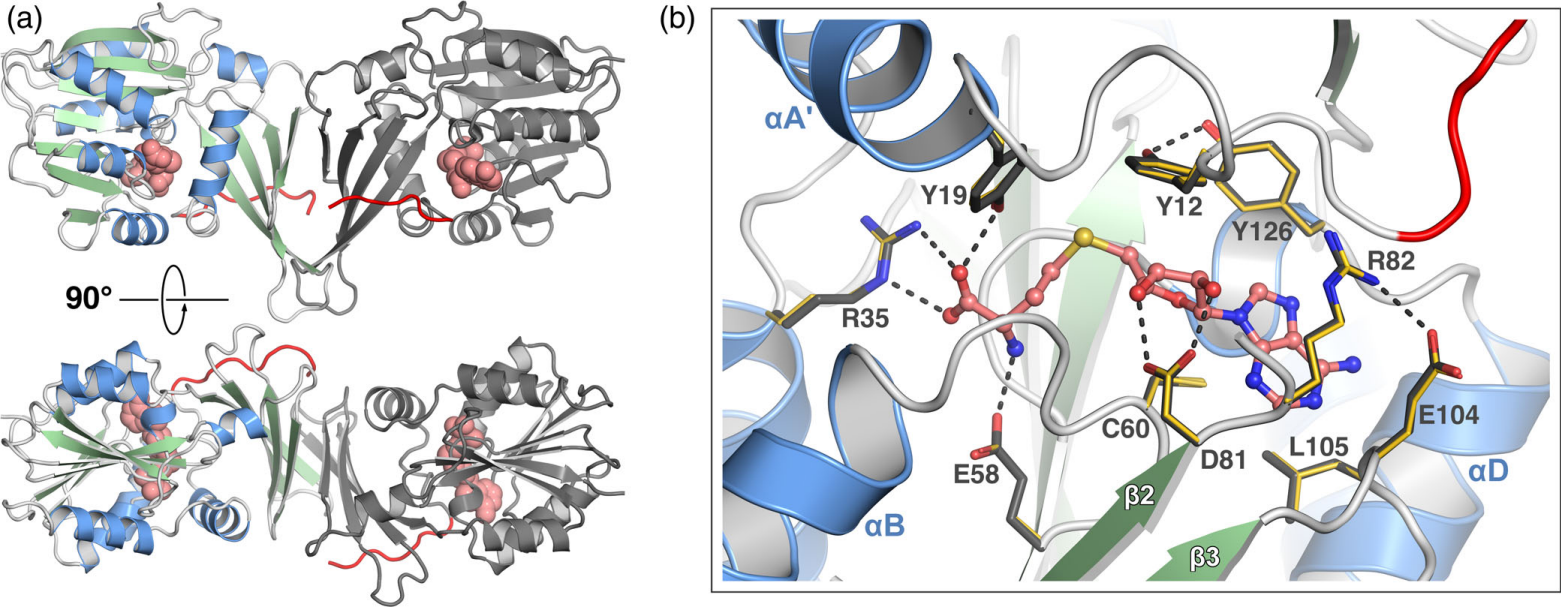
X‐ray crystal structure of SeMT. (a) The overall fold and quaternary structure of SeMT closely resemble those found for indole‐targeting methyltransferases PsmD and SgMT. The N‐terminus (residues preceding D10, discussed below) is highlighted in red, the cofactor (SAH) is shown in space‐filling representation. (b) Superimposition reveals strict conservation of the cofactor binding pocket between SeMT (side chains in dark gray) and SgMT (side chains in gold).

Crystals of SeMT belong to orthorhombic space group C222_1_ and contain two copies of the protomer per asymmetric unit. As expected from the amino acid sequence similarity, SeMT features the same overall architecture as found for indole C3‐methyltransferases SgMT (Haase et al. 2025) and PsmD (Amariei et al. 2022), with the putative catalytic cavity located at the interface of a Rossmann‐type α/β domain and an all‐β cap domain (Figure 3a). The two chains in the asymmetric unit are highly similar, featuring root‐mean‐square (RMS) distances of 0.55 Å both for 264 matching Cα atoms and for 1060 main‐chain atoms (least‐squares alignment using COOT (Emsley et al. 2010), using the prevalent conformer for residues with alternative conformations). In agreement with our previous findings with SgMT and PsmD, this dimeric assembly is considered the biologically significant unit. In fact, superimposition of the SeMT dimer with chains A and B of SgMT (PDB‐ID 9GDJ) using GESAMT (Krissinel 2012) yields an overall RMS distance of 1.37 Å for 506 aligned Cα atoms, compared to an average pairwise RMS distance of 0.83 Å when comparing individual chains of the two complexes. The symmetric interaction via the two cap domains constitutes the most prominent protein–protein interface in either crystal structure, according to PISA analysis (Krissinel and Henrick 2007), with buried solvent‐accessible surface areas of 717 and 924 Å^2^ for the A‐B interfaces of SeMT and SgMT, respectively. These observations attest to the strong conservation of both the protomer structure and the biological assembly.

In accordance with these overall similarities, the precise arrangement of the active center in SeMT is reminiscent of indole‐targeting methyltransferases SgMT and PsmD in many respects. In particular, the demethylated cofactor SAH (included in the sample for crystallization) is found embraced by a binding pocket that is virtually identical to the one found in those enzymes (Figure 3b); this includes R82 and C60 essentially sandwiching the planar adenine ring, D81, which is hydrogen‐bonded to both hydroxyls of the ribose moiety, as well as E58 and R35 forming salt bridges with the homocysteine ammonium and carboxylate groups, respectively. Moreover, the homocysteine β carbon is in tight van‐der‐Waals contact with G62, which is part of a glycine‐rich motif (GTG in all three proteins considered here) connecting strand β1 to the subsequent helix and representing an ancient signature of the Rossmann fold. Finally, the characteristic tyrosine side chains covering the cofactor (Y12, Y19, Y126) as well as the histidine‐acidic residue dyad thought to participate in catalysis (H218, E216) are retained. Overall, this conservation of key features deemed essential for methyl transfer strongly suggests that SeMT is an active enzyme.

### 2.3 Effects of amino acid exchanges on catalytic activity

**FIGURE 4:**
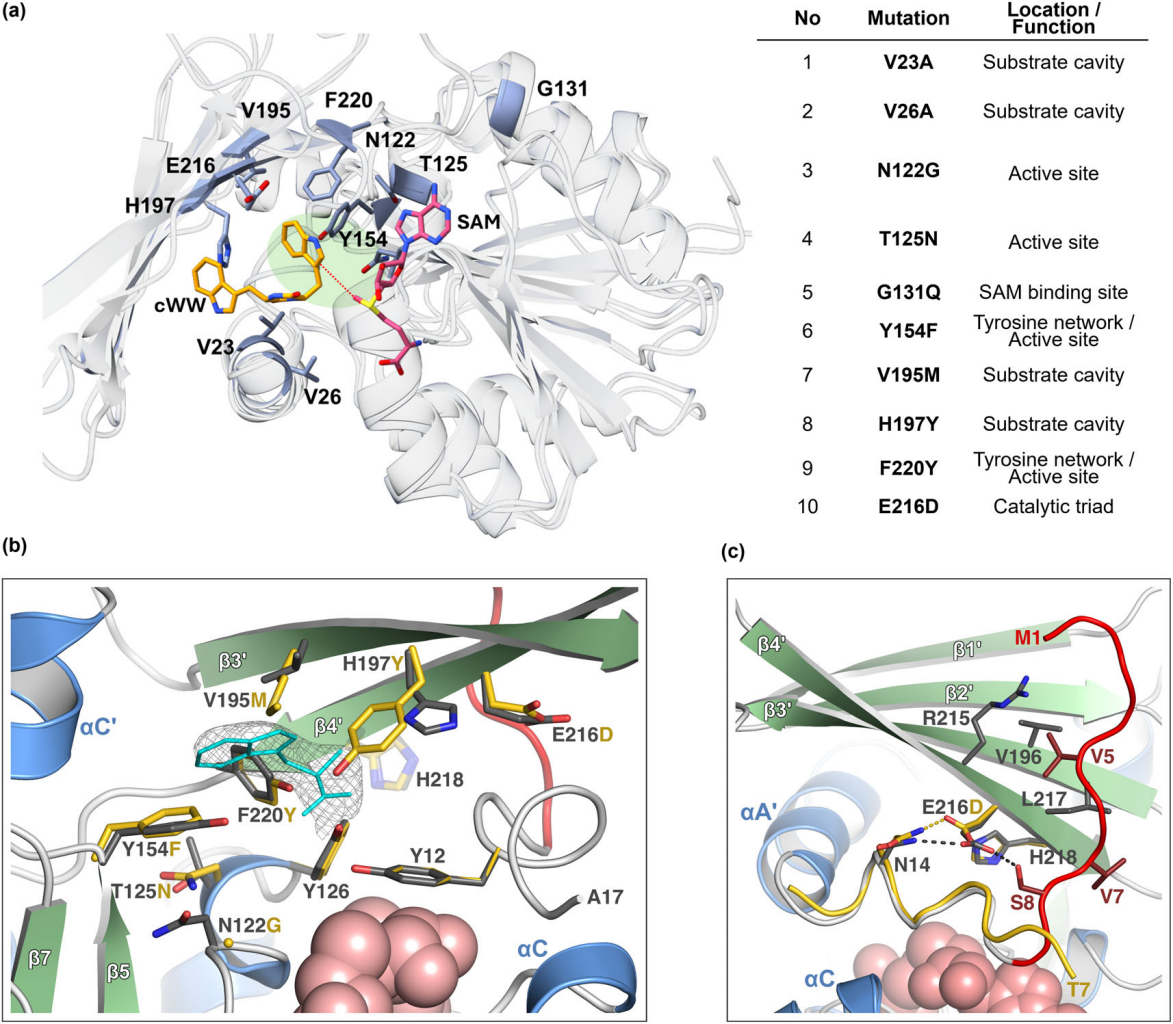
Differences between SeMT and SgMT proteins affecting catalytic activity. (a) Overall superimposition of the two structures and the location/function of SeMT residues investigated in our mutagenesis study (blue). The cofactor SAM is highlighted in pink, the cWW substrate, which was docked in SgMT in our previous work (Haase et al. 2025), in orange. The cWW molecule contains two indole moieties, with one positioned in the active site in close proximity to the cofactor (highlighted in green). (b) Close‐up view of residues lining the substrate binding cavities of SeMT (side chains in dark gray) and SgMT (side chains in gold). The majority of amino acid exchanges investigated in this study are indicated (V23A, V26A, and G131Q omitted for clarity). Electron density (2*mF*_*o*_‐*DF*_*c*_ synthesis, contoured at 0.9 sigma) signifies an unidentified ligand, which can be reasonably approximated by a small indole‐containing compound (cyan wireframe). (c) In contrast to SgMT (coil in gold), SeMT adopts a bent conformation in its N‐terminal segment (residues 1–9 colored red) stabilized by hydrogen bonding interactions of E216 as well as hydrophobic contacts.

While SeMT clearly provides the basic machinery required for methyl transfer, its target preferences should be dominated by the cavity accommodating the substrate. Indeed, while the catalytic cavity of SeMT resembles its counterpart in SgMT with respect to size (417 vs. 378 Å^3^, determined with the cofactor in place, average of chains A and B in either case) and overall character (hydrophobic patches dominated by aromatic side chains, interspersed with polar groups), residues at individual positions often differ notably in size and polarity (Figure 4b). Similar considerations apply when comparing SeMT with the more distantly related PsmD. Overall, these observations strongly suggest that SeMT has evolved to process a different class of substrates, in accordance with our activity data (Figure 2).

Nine specific residues, chosen based on their conservation between StspM1 and SgMT and their proximity to the active center, were selected for further analysis (Figures 4a and S1). We reasoned that by mutagenesis at these positions, yielding an active center resembling that of SgMT, SeMT might acquire activity towards the cWW substrate. The residues of interest mostly surround the putative cWW binding site, except for G131, which corresponds to a glutamine in both StspM1 and SgMT and is located in the first turn of helix αD, at the bottom level of the cofactor binding cleft. While residue 131 is not in direct contact with SAM, the character of its side chain can be expected to influence solvation in the vicinity of the cofactor adenine moiety. In fact, L127 (at the beginning of helix αD) is mostly shielded by Q131 in SgMT, whereas the aliphatic side chain appears solvent‐exposed in SeMT, forming a shallow hydrophobic patch together with G131 and P128. The V23A and V26A mutations primarily create additional space in the active center due to effective truncation of side chains. Mutations V195M and H197Y not only adjust steric properties but also introduce different polar interactions. Considering the positioning of the cWW substrate previously inferred from docking and molecular dynamics simulations (Haase et al. 2025), these four residues are located near the DKP ring, rather than the indole ring where the actual methylation occurs. In contrast, the T125N and N122G mutations are located closer to the methylation area. Combining exchanges Y154F and F220Y is equivalent to switching the position of a hydroxyl group in the active site. In StspM1, F220 of SeMT aligns with Y223, a residue essential for enzyme activity by forming a hydrogen bond with the substrate's reactive indole nitrogen (Haase et al. 2023). In SgMT, homologous Y222 is thought to play a key role in accurately positioning Y126, which is part of the catalytic triad, for effective catalysis (Haase et al. 2025).

**FIGURE 5:**
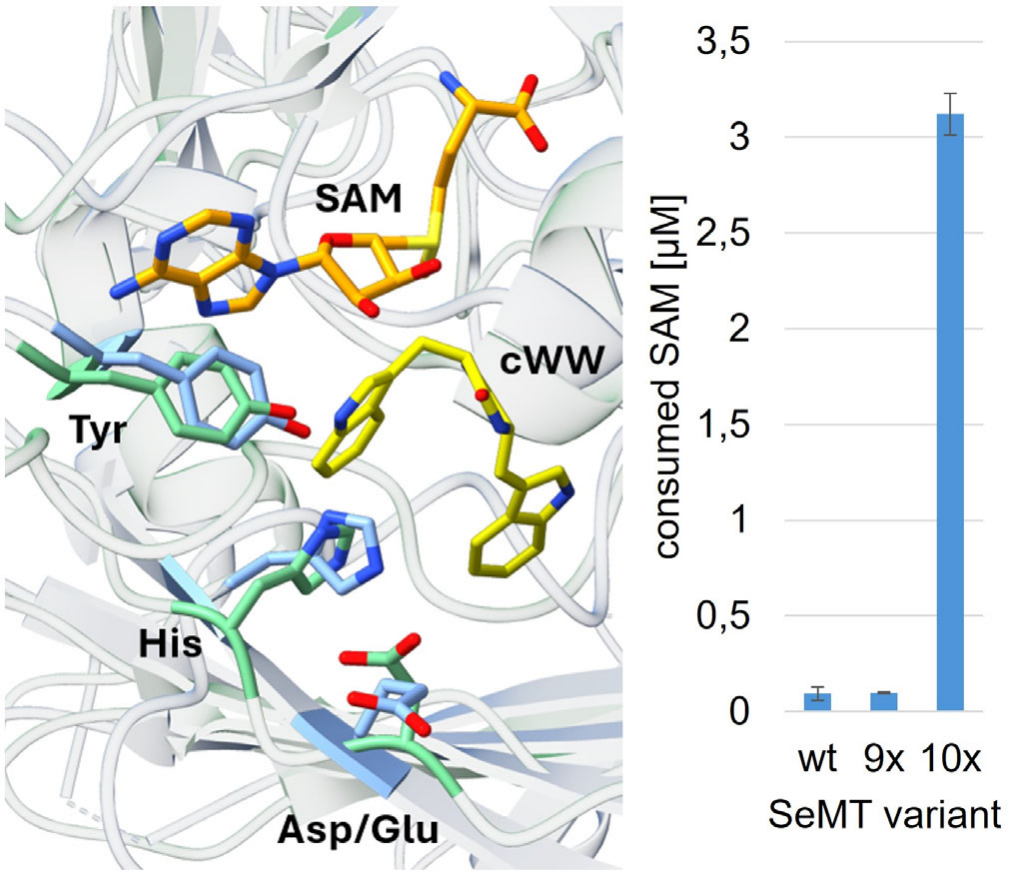
Catalytic centers of SeMT and SgMT, with amino acid residues of the catalytic triads colored blue and green, respectively. The cofactor, shown here as SAM, is highlighted in orange, the substrate cWW (docked position in SgMT) in yellow. The activities of wild‐type SeMT as well as its 9× and 10× mutants have been determined at 45°C after 15 min reaction time using cWW as substrate. The activity was measured with the MTase Glo Assay (Promega), which detects the consumption of SAM.

The resulting 9× mutant was tested again for SAM conversion with the cWW substrate; unexpectedly, no increased activity was detected at 45°C, which is the optimal temperature for conversions with SgMT (Figure 5). Searching for further potential bottlenecks of catalytic efficiency, the acidic residue on strand β4′, which is supposed to participate in the catalytic triad, caught our attention. In SeMT this residue is a glutamate (E216), whereas SgMT and StspM1 have an aspartate at the same position. While glutamate and aspartate feature similar chemical characteristics owing to their carboxylic acid groups, the difference in chain length may still affect enzymatic activity. In fact, when the corresponding aspartate in SgMT (D218) was mutated to glutamate, enzyme activity was significantly reduced (Haase et al. 2025). Conversely, the indole C3‐methyltransferase PsmD from *Streptomyces griseofuscus*, which participates in the biosynthesis of physostigmine, naturally contains a glutamate at this position, and in this case, replacement with aspartate led to a substantial decrease in activity (Amariei et al. 2022). Recognizing the importance of this residue, the glutamate in SeMT was replaced with an aspartate as a tenth mutation. Indeed, this 10× mutant demonstrated activity that was 33 times higher than that of the wild‐type enzyme (Figure 5).

**FIGURE 6:**
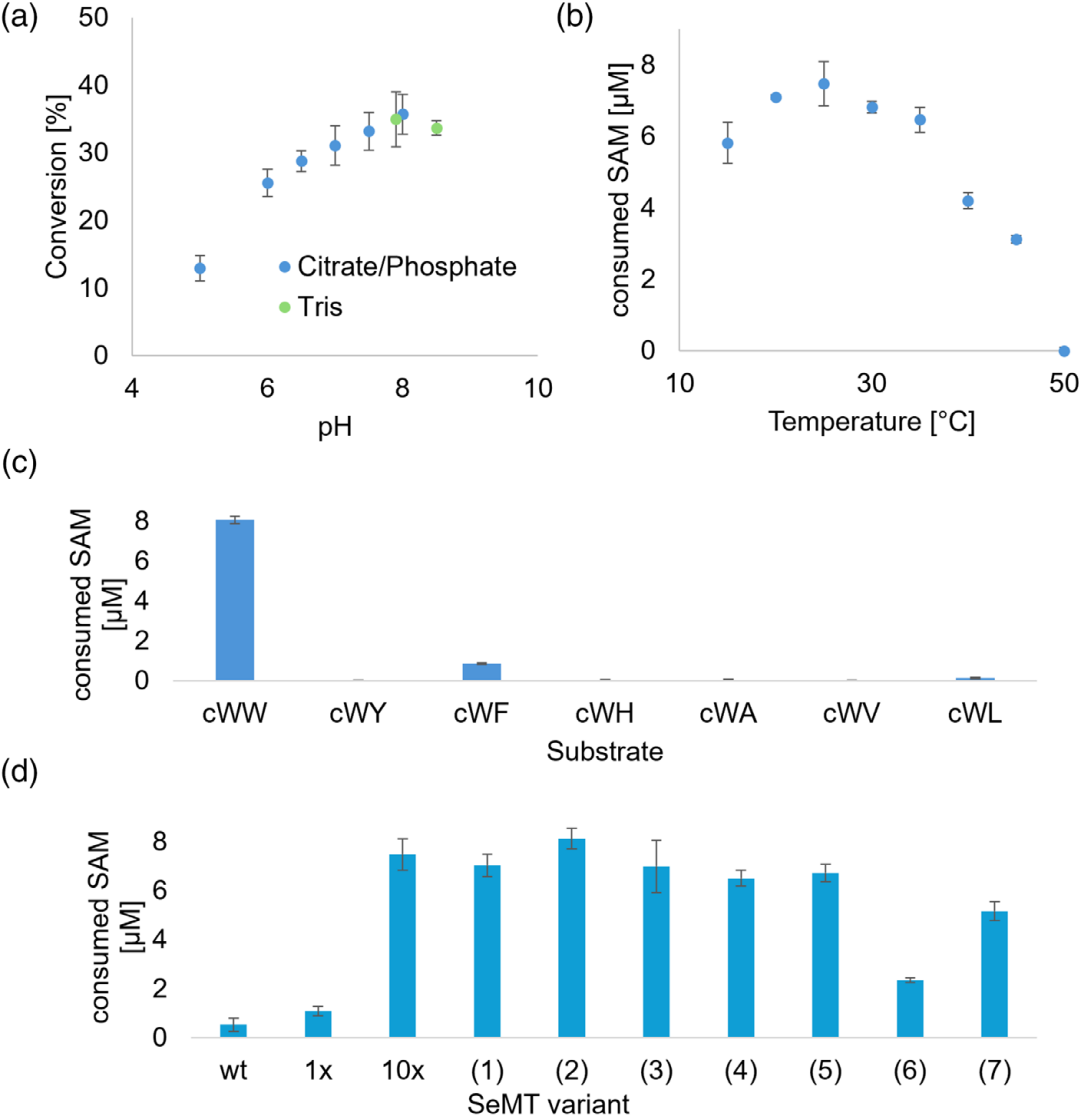
Biochemical characterization of the SeMT 10× mutant (see Figure 4a). (a) pH profile measured via HPLC. (b) Temperature profile measured with the MTase‐Glo Assay. (c) Substrate scope measured with the Glo Assay. (d) Activities of different SeMT variants measured with the Glo Assay using cWW as substrate. The 10× mutant contains all 10 mutations from Figure 4a, the 1× mutant contains only the E216D mutation. The other variants are based on the 10× mutant, with the annotated mutations being reversed toward the wild type, leading to 9× or 8× mutants. (1) V23A + V26A, (2) N122G + T125N, (3) G131Q, (4) Y154F, (5) V195M + V195Y, (6) E216D, (7) F220Y. The methylation reactions measured with HPLC were carried out for 4:45 h, and the reactions measured with the Glo Assay for 15 min at 25°C.

The active 10× mutant was further characterized (Figure 6a–c). Beyond the strongly preferred cWW, the substrate acceptance profile of SeMT was found to be similar to that of SgMT and StspM1 (Table S5). The same is true for the pH optimum (determined using HPLC; see section 5); the temperature optimum, however, differed by as much as 20°C, as the highest conversion for SeMT occurred at 25°C, whereas the optimum for both SgMT and StspM1 was 45°C. Based on these findings, the optimal conditions for SeMT (pH 8, 25°C) were used in subsequent experiments. Overall, our observations indicate that the enzymatic activity, and hence most likely the catalytic mechanism, applied by SgMT and StspM1 has been successfully established in the 10× mutant of SeMT.

The intriguing effect of the E216D exchange, with nine other mutations already in place, prompted us to re‐evaluate the contributions of individual modifications. To this end, the mutations were reverted to the respective wild‐type residues one at a time, with the exception of the V23‐V26, N122‐T125, and V195‐H197 pairs that were reverted together due to their proximity. Enzyme activity was measured using purified enzyme with the cWW substrate at the optimal temperature of 25°C (Figure S3 and Table S6). Under these conditions, the wild‐type enzyme remained nearly inactive, whereas all mutants retained significant activity. As expected, the strongest loss of function was observed upon reversal of the E216D exchange, yielding the original 9× mutant, but even this one preserved measurable activity at 25°C, which was not the case at 45°C in previous experiments (Figure 6d). It is important to note that, while the E216D mutation provides the largest individual contribution in the context of the 10× mutant, it is unable to effect a large increase in activity with the cWW substrate on its own, pointing to strong synergies with the set of nine, designed for priming the substrate cavity.

**FIGURE 7:**
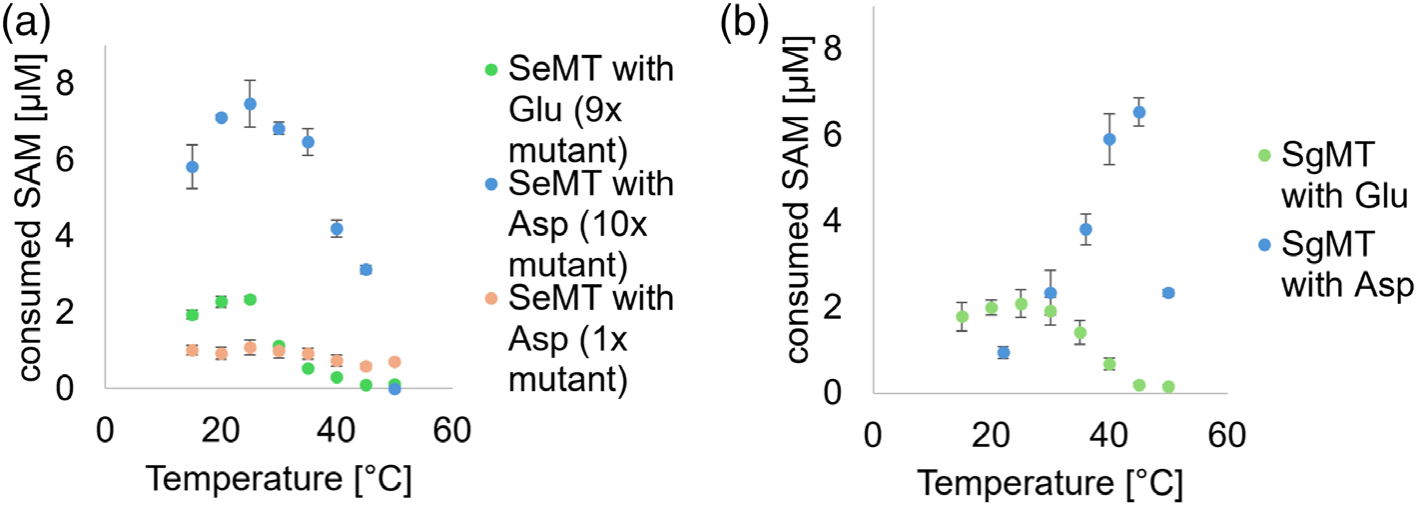
Temperature‐activity profiles of SeMT, SgMT, and variants thereof, determined at enzyme concentrations of 10 μM for SeMT and 3 μM for SgMT after incubation at the desired temperature for 15 min. (a) The 9× SeMT mutant (green) contains the first nine exchanges listed in Figure 4a, the 10× mutant (blue) contains additionally the E216D mutation. The single mutant (orange) is based on the wild type with only the E216D mutation. (b) SgMT wild type (blue) (Haase et al. 2025) and its single mutant D218E (green).

The 20‐K difference in optimal conversion temperature observed between SgMT and re‐engineered SeMT was remarkable in light of the fact that (i) both proteins feature highly conserved tertiary and quaternary structures and (ii) their respective host organisms are adapted to similar ambient temperatures. To clarify the impact of residue 216 (218 in SgMT) on the enzyme's temperature‐activity profile, the temperature screening was additionally conducted for the 9× mutant of SeMT (containing E216), as well as for both the wild‐type SgMT (with the native D218) (Haase et al. 2025) and an SgMT mutant with glutamate at this position (Figure 7). Both SeMT variants showed an optimal temperature of 25°C, indicating that this feature is intrinsic to the 9× mutant in conjunction with the cWW substrate and not connected to the activating effect of the E216D exchange. In stark contrast, the reverse modification (D218E) in SgMT shifted the temperature optimum from 45 to 25°C, along with a reduction in overall activity. It is interesting to note that the 9× mutant of SeMT and the D218E variant of SgMT—both containing a cWW‐adapted (engineered or native) catalytic cavity and a glutamic acid in the catalytic triad—display an almost identical temperature profile (green symbols). In either case, the presence of aspartic acid at position 216/218 effects a threefold to fourfold increase in maximum activity which, in the case of SgMT (and possibly StspM1), is connected with a considerable shift of the temperature optimum. In order to assess the impact of amino acid exchanges on overall protein stability, thermal unfolding of the SeMT wild type, SeMT 10×mutant, SgMT wild type, and SgMT D218E mutant was recorded by differential scanning fluorometry (DSF), monitoring intrinsic tryptophan and tyrosine fluorescence (350/330 nm emission ratio) in response to increasing temperature (Figure S5). The SeMT wild type protein exhibited an inflection point in the melting curve at 43.7°C, which is around 2°C higher than the major transition temperature of the 10× mutant (41.5°C). Remarkably, in the case of the mutant the fluorescence ratio starts rising way below 30°C, and its first derivative suggests at least a second discrete transition occurring at about 33°C. In comparison, the SgMT variants demonstrated higher thermal stability, with inflection points at 49.7°C for the wild type and 51.2°C for the D218E mutant. Overall, the mutations appear to have only a minor impact on enzyme thermostability (Table S7).

## 3 DISCUSSION

This study is embedded in an ongoing effort aiming at the adoption of biological catalysts for challenging synthetic applications, with a focus on indole C3 alkylation. Given that natural enzymes, even if catalyzing the same basic reaction, can be expected to feature a wide variety of adaptations both to details of individual substrates and to environmental conditions, a one‐fits‐all strategy is unlikely to be appropriate since individual scaffolds will inevitably differ in their adaptability to a specific process. Having characterized methyltransferases PsmD and SgMT/StspM1, which are naturally involved in the synthesis of different HPI‐containing products, we therefore set out to search for novel candidates featuring unique properties. The SeMT protein from *Saccharopolyspora erythraea* investigated in this work features significant sequence and structural similarity to the above enzymes, yet its natural substrate is clearly distinct. Despite our efforts in identifying a bona fide substrate for wild‐type SeMT (investigation of its genetic environment, testing of candidate compounds for turnover), its role in the host organism remains elusive at this time. Notwithstanding, rational engineering enabled us to convert this putative enzyme into an active catalyst processing an indole‐substituted diketopiperazine (cWW). Intriguingly, optimum activity was found to specifically require an aspartyl residue at position 216 instead of the natural glutamyl, in addition to a set of mutations adopting the catalytic cavity to the substrate of choice. While a preference for either acidic residue at this position is not unprecedented, its structural underpinnings have remained obscure. Our SeMT crystal structure suggests a potential explanation for the striking effects of the E‐D exchange on the enzyme's activity profile. Analogous to PsmD and SgMT, the N‐terminal segment extending the canonical Rossmann fold in SeMT plays an important role in confinement of the catalytic cavity. Preceding a regular and stable α‐helix (αA′), it features a more irregular terminal stretch we have termed the lid for its presumed role in controlling access to the active center during the catalytic cycle. In contrast to SgMT, this segment in SeMT forms a 90° bend centered on S9, causing the N‐terminus to extend towards the edge strand (β4′) of the β‐cap domain. This conformation, which is observed consistently in both chains of the crystal asymmetric unit and is thus unlikely to represent a lattice packing artifact, is stabilized by hydrophobic interactions of V5 and V7 with V196, L217 and the aliphatic moiety of R215. Intriguingly, E216 also contributes to this feature by hydrogen bonding to both S8 and N14, that is, by bridging residues on either side of the kink (Figure 4c). Its counterpart D218 in SgMT does form the analogous bond to N13 but lacks an N‐terminal interaction partner; in fact, residues preceding P8 appear disordered in this case. We hypothesize that the additional interactions involving the N‐terminal stretch will restrain certain aspects of dynamics, first and foremost the mobility of the lid itself, thus limiting access of bulky substrate molecules to the catalytic cavity, but possibly extending to concerted movements of the β‐cap domain, which is effectively clamped to the Rossmann fold. The E216D exchange in SeMT still appears compatible with hydrogen bonding to N14, whereas the link to S8 will probably be lost, thus destabilizing the auto‐inhibitory interactions of the N‐terminal stretch and allowing cWW to enter the catalytic site. Obviously, efficient processing of this particular substrate additionally requires adjustments to the substrate cavity (as in the 9× mutant) to accommodate and pre‐orient the compound for catalysis (Figure 6d).

It is interesting to note that, in the case of SeMT, the E‐D exchange appears to switch the protein into a higher‐activity state without altering the temperature optimum. In our view, this can be rationalized by recognizing that catalytic activity is modulated by protein dynamics on different structural levels. Specifically, a loss of interactions in the N‐terminal stretch, facilitating a (sub)domain level motion presumably governing communication of the catalytic cavity with the exterior, will not necessarily affect the more localized dynamics of the core catalytic residues. By facilitating diffusive exchange, the effect of the E216D mutation may hence conceptually resemble an increase in substrate concentration (and/or a decrease in product concentration), leaving basic characteristics of catalysis, such as the temperature optimum, unchanged.

Could the reverse mechanism underlie the reduction of activity observed for the D218E variant of SgMT? Indeed, SgMT contains a threonine residue (T7) analogous to S8 in SeMT, and the adjacent valine (one of the hydrophobic anchors in SeMT) is conserved as well; it is therefore tempting to speculate that the presence of a glutamate side chain might restrain the enzyme in similar ways as outlined above for native SeMT. Notably, this model is consistent with our DSF data indicating a moderately stabilizing effect of the D218E exchange. It does not, however, explain the dramatic down‐shift in the temperature optimum of SgMT‐D218E, which happens to coincide with the optimum observed for the re‐engineered SeMT with or without the E‐D exchange. This observation implies that in SgMT, the (non‐native) glutamyl residue not only is less favorable per se, but becomes even more disadvantageous to activity as temperature is increased beyond 25°C. While the underlying mechanism is not immediately obvious from the SgMT crystal structure, one potential hypothesis relates to the differential side chain entropies of aspartic and glutamic acid; these would translate into a higher overall mobility of the latter, possibly with more pronounced loss of electrostatic stabilization of the catalytic histidine as temperature is increased.

Although significant activity of SeMT towards cWW could be established through the introduction of 10 mutations, its overall activity at its optimal temperature of 25°C remains three times lower than that of SgMT at 45°C (Haase et al. 2025) (note the differences in enzyme concentration used in the assays: 10 μM for SeMT and 3 μM for SgMT). On the other hand, considering that this difference is less than expected from basic kinetics theory (van't Hoff's approximation predicts a factor of 4–9), the temperature‐corrected activity of the SeMT 10× mutant would actually appear to be higher, but this advantage cannot be exploited at elevated temperatures probably due to the limits imposed by protein stability. Indeed, the major inflection point of thermal unfolding (41.5°C; Table S7) is in good agreement with the temperature at which activity has declined to 50% of its optimum at 25°C (Figure 7a). Conversely, SgMT's methyltransferase activity drops significantly at lower temperatures, indeed falling short of the SeMT 10× mutant at 25°C, but is stabilized by the D218E exchange. It follows from the above that both the 10× mutant of SeMT and the single D218E mutant of SgMT may serve as advantageous catalyst options for biosynthetic reactions conducted at temperatures lower than 30°C.

## 4 CONCLUSION

In this study, a hitherto uncharacterized methyltransferase (SeMT) from the erythromycin‐producing actinomycete *Saccharopolyspora erythraea* was subjected to an in‐depth structural and enzymological investigation, with the aim of extending the toolbox of catalytic scaffolds amenable to adaptation for biomimetic synthesis. Its three‐dimensional structure determined through X‐ray crystallography not only guided the rational re‐design of the catalytic center to accommodate a non‐native substrate but also helped rationalize the striking effect of an additional mutation (E216D) boosting catalytic efficiency. The latter is particularly significant because this acidic residue, while conserved by its nature, was found to participate in partly non‐conserved interactions across related methyltransferases, controlling their activities in an intricate manner. We are confident that these insights will prove helpful in future endeavors aiming at rational engineering of native enzymes for application as biocatalysts, which includes tuning of substrate acceptance and temperature profiles.

## 5 MATERIALS AND METHODS

### 5.1 Protein sequences and vectors

The gene sequences for the methyltransferase SeMT and its 9× mutant (refer to Data S1 for details) were codon‐harmonized and synthetically constructed in a pET28a(+) vector (GenScript, USA).

### 5.2 Bacterial strains and media

*Escherichia coli* BL21(DE3) was used for protein expression, while *E. coli* DH5α was chosen for plasmid amplification. LB (lysogeny broth) liquid medium (10 g/L tryptone, 5 g/L yeast extract, 2 g/L sodium chloride) was used for pre‐cultures, and TB (terrific broth) liquid medium (Carl Roth, Karlsruhe, Germany: 12 g/L casein, 24 g/L yeast extract, 12.54 g/L K₂HPO₄, 2.3 g/L KH₂PO₄, 4 mL/L glycerol) with kanamycin (final concentration of 100 μg/mL) was used for main cultures. All media were prepared with distilled water and sterilized by autoclaving.

### 5.3 Transformation

Competent cells were transformed with the desired plasmid via heat shock. Specifically, 100 ng of plasmid DNA was added to 100 μL of competent cells and incubated on ice for 30 min. The mixture was then heat‐shocked in a 42°C water bath for 90 s. Following this, 700 μL of LB medium was added, and the cells were agitated on an overhead shaker at 37°C for 1 h. The transformed cells were then centrifuged at 5000*g* for 2 min to form a pellet, which was resuspended in 100 μL of LB medium. Finally, the resuspended cells were plated on LB agar plates containing kanamycin and incubated overnight at 37°C.

### 5.4 Protein expression

A pre‐culture was prepared by inoculating 5 mL of LB medium containing kanamycin with a single colony from the transformed cells, followed by incubation at 37°C for 16 h. This preculture was subsequently used to inoculate the main culture (500 mL TB medium) at a 1:100 dilution. The main culture was grown at 37°C with gentle shaking until reaching an OD_600_ of 0.5. Protein expression was then induced by adding isopropyl β‐D‐thiogalactopyranoside to a final concentration of 100 μM. Following an incubation at 25°C for 20 h, the cells were harvested by centrifugation at 7000 × *g*! for 35 min at 4°C. The resulting cell pellet was stored at −20°C for further use.

### 5.5 Enzyme purification

A 200‐mg sample of cells was resuspended in 1 mL of potassium phosphate (KP_i_) buffer (50 mM, pH 8) and lysed using the glass bead (0.2 mm) cell disruption method for 10 min at 30 Hz in a swing mill (Retsch, Type MM400). The lysate was collected after centrifuging at 11,000*g* for 30 min and then incubated with 100 μL of pre‐washed Ni‐NTA agarose beads by gentle inversion on ice for 40 min. The beads were washed twice with 1 mL of 50 mM KP_i_ buffer containing 80 mM imidazole, pH 7.5 to remove non‐specifically bound proteins. The target protein, SeMT, was then eluted with 500 μL of elution buffer (50 mM KP_i_, 160 mM imidazole, pH 7.5). For buffer exchange and protein concentration, Vivaspin 500 centrifugal concentrators (10,000 MWCO PES, Sartorius Stedim Biotech) were used.

For a large‐scale enzyme purification, the cells were resuspended in KP_i_ buffer (50 mM, pH 8) at a concentration of 0.2 g/mL. Cell lysis was performed by sonication with an ultrasonic cell disruptor (Branson Sonifier II “Modell W‐250,” Heinemann) two times for 10 min (amplitude of 35%–40%). The cleared lysate was loaded on a Ni‐NTA affinity chromatography column (5 mL HiTrap HP column, Bio‐Rad) for enzyme purification. After a washing step with 15 mL 50 mM KP_i_, 80 mM imidazole (pH 7.5), SeMT was eluted with 15 mL of the elution buffer (50 mM KP_i_, 160 mM imidazole, pH 7.5). For buffer exchange and protein concentration, Vivaspin 500 centrifugal concentrators (10,000 MWCO PES, Sartorius Stedim Biotech) were used.

### 5.6 Mutagenesis

The desired mutations were introduced at the center of the respective forward primers. The PCR mixture was prepared following Table S1, and the protocol in Table S2 was applied. To introduce the tenth mutation (E216D), the 9× mutated SeMT gene in pET28a(+) was used as the template. Variants with certain mutations reversed were generated based on the 10× mutated SeMT gene in pET28a(+). After PCR, products were digested with DpnI for 1 h at 37°C to remove the template DNA and then analyzed by 1% agarose gel electrophoresis with Midori Green stain. Following gel purification, the products were ligated with T4 DNA ligase in the presence of 6% (w/v) PEG 4000. Subsequently, 20 μL of the ligated product was used to transform chemically competent *E. coli* DH5α cells via heat shock. Plasmids were isolated using a commercial kit (Innuprep, Analytik Jena), and the SeMT gene was sequenced to confirm the desired modification.

### 5.7 Determination of methyltransferase activity

For most applications described in this work, the catalytic activity of methyltransferase variants was measured using the commercially available MTase‐Glo™ Methyltransferase Assay (Promega), following the manufacturer's instructions. Luminescence readings were taken with an Infinite plate reader (Tecan). All reactions were performed in triplicate in a 96‐well plate (Nunc™; polystyrene, flat bottom, white). Each reaction contained 10 μM enzyme (SeMT variants) or 3 μM enzyme (StspM1 and SgMT variants), with final concentrations of 25 μM for both SAM and the respective substrate. Reaction mixtures were incubated at the desired temperature for 15 min and stopped by adding 0.5% trifluoroacetic acid (TFA).

When assessing the pH dependence of enzyme activity, an HPLC‐based assay was applied: A reaction mixture was prepared by combining 1 mM cWW substrate, 1 mM SAM, and 50 μg of enzyme in reaction buffer to a final volume of 100 μL. The mixture was incubated at 30°C with shaking at 300 rpm for 4 h and 45 min. The reaction was then quenched by adding TFA to a final concentration of 1%. Conversion rates were analyzed using HPLC. RP‐HPLC was performed using a Jasco HPLC system (pump: PU‐4180, thermostat: CO‐2060Plus, autosampler: AS‐4050). The stationary phase consisted of a Hyperclone column (5 μm ODS C18, 125 × 4 mm, 120 Å, Phenomenex). The mobile phase components were (A) acetonitrile with 0.1% (v/v) formic acid and (B) water with 0.1% (v/v) formic acid. The gradient elution program was as follows: 0–2 min: 10% A, 90% B; 2–15 min: 10% A to 50% A; 15–25 min: 50% A to 95% A; 25–30 min: 95% A, 5% B; 30–35 min: 10% A, 90% B. The flow rate was set to 0.8 mL/min, with a column temperature of 25°C, and detection was carried out at 284 nm.

### 5.8 X‐ray crystallography

Wild‐type SeMT was crystallized using the sitting‐drop vapor diffusion method, using robotic systems Freedom Evo (Tecan; Männedorf, Switzerland) and Mosquito LCP (SPT Labtech; Melbourn, UK) with commercially available screening sets. Initial protein crystals were observed with reservoir solutions containing either 2.0 M ammonium sulfate or 1.0 M lithium sulfate, 0.5 M ammonium sulfate, 0.1 M sodium citrate pH 5.6, which were subjected to matrix optimization and additive screening. The diffraction‐quality sample used for structure determination in this study was obtained with 0.9 M lithium sulfate, 0.45 M ammonium sulfate, 0.09 M sodium citrate pH 5.6, and 3% (w/v) 1,6‐diaminohexane in the reservoir and a sample containing 8.2 mg/mL SeMT and 2 mM SAH in 10 mM Tris–HCl pH 7.0, 100 mM NaCl, and 4% (v/v) DMSO. While harvesting the crystal, it was shortly submerged in perfluoropolyether (Hampton Research; Aliso Viejo, USA) for cryoprotection. 100‐K diffraction data (https://doi.org/10.15151/ESRF-DC-2014789904) were recorded at the European Synchrotron Radiation Facility (ESRF; Grenoble, France), using beamline ID30B equipped with an EIGER2 Si 9M detector (DECTRIS; Baden‐Daettwil, Switzerland) and tuned to an X‐ray wavelength of 0.873 Å. Following integration and scaling of data using the XDS suite (Kabsch 2010), initial coordinates for SeMT containing its cofactor SAH were obtained by molecular replacement using MOLREP (Vagin and Teplyakov 2010) with the crystal structure of the methyltransferase PsmD from *Streptomyces griseofuscus* (PDB‐ID 7ZKH) as a search model. After a round of automated rebuilding in phenix.autobuild (Terwilliger et al. 2008), the model was subjected to reciprocal space refinement in phenix.refine (Liebschner et al. 2019) alternating with interactive rebuilding in COOT (Emsley et al. 2010). Atomic displacement was parameterized using isotropic B‐factors combined with a TLS model, based on the Hamilton R‐factor ratio test implemented in PDB‐REDO (Joosten et al. 2014). Assessment via MolProbity (Williams et al. 2018) and the wwPDB validation system (https://validate-rcsb-1.wwpdb.org/) confirmed good model geometry; for full data collection and refinement statistics refer to Table S3. Coordinates and structure factor amplitudes have been deposited in the wwPDB with PDB code 9ICZ (https://doi.org/10.2210/pdb9icz/pdb).

Illustrations of protein structures were prepared using ChimeraX (Meng et al. 2023) or open‐source PyMOL (https://github.com/schrodinger/pymol-open-source). Cavities were calculated using the CavitOmiX plugin (Innophore GmbH), which makes use of the hydrophobicity module of VASCo (Steinkellner et al. 2009) as well as a modified LIGSITE (Hendlich et al. 1997) algorithm.

### 5.9 Thermostability assay

Thermostability measurements were performed in triplicate using the Prometheus NT Plex DSF instrument (NanoTemper Technologies; Munich, Germany). Enzyme samples were prepared at a concentration of 1 mg/mL in KP_i_ buffer (50 mM, pH 8). The temperature was increased from 20 to 60°C at a rate of 2°C/min. The fluorescence ratio at 350 nm/330 nm was monitored (280 nm excitation wavelength), and the inflection point of the resulting melting curve was determined via the first derivative.

## AUTHOR CONTRIBUTIONS

**Mona Haase:** Conceptualization; methodology; writing – original draft; formal analysis; data curation; validation; investigation; visualization. **Oliver H. Weiergräber:** Investigation; formal analysis; writing – original draft; supervision; resources; methodology; validation. **Jörg Pietruszka:** Conceptualization; supervision; funding acquisition; writing – review and editing; project administration; resources.

## Supporting information


**Data S1.** Supporting Information.

## Data Availability

The data that support the findings of this study are available in Supporting Information of this article.

## Footnotes

* We are using this term to emphasize that the protein is evolutionarily designed for a different function.
