# Supplementary material for: Re‐engineering a transferase scaffold for indole C3 methylation in diketopiperazines
Source: Protein Sci. 2025 Aug 28;34(9):e70254. doi: 10.1002/pro.70254 (PMC12394179; doi:10.1002/pro.70254)
Supplement: Supplementary file 1 — Data S1. Supporting Information. [file PRO-34-e70254-s001.pdf]

Re-engineering a transferase scaffold for indole C3 methylation in diketopiperazines

Mona Haase[1], Oliver H. Weiergräber[2,#], Jörg Pietruszka[1,3,$]

[1] Institute of Bioorganic Chemistry & Bioeconomy Science Center (BioSC),
Heinrich Heine University Düsseldorf in Forschungszentrum Jülich,
52426 Jülich, Germany

[2] Institute of Biological Information Processing (IBI-7: Structural Biochemistry),
Forschungszentrum Jülich, 52425 Jülich, Germany

[3] Institute of Bio- and Geosciences (IBG-1: Bioorganic Chemistry)
Forschungszentrum Jülich, 52425 Jülich, Germany

[#] Oliver H. Weiergräber, phone: +49 2461 61-2028, fax: +49 2461 61-9540,
e-mail: o.h.weiergraeber@fz-juelich.de

[$] Jörg Pietruszka, phone: +49 2461 61-4158, fax: +49 2461 61-6196,
e-mail: j.pietruszka@fz-juelich.de

Supporting Information

Table of Content

Protein and gene sequences S2

PCR S4

SDS-Gels S5

Crystallographic Data S6

Activity Data S7

Protein and gene sequences

Terminal residues not present in the native sequence (UniProt ID A4FFC0) are underlined.

SeMT_wt

MGHMTVDVPVSSDPYANLAASYDRLVDWVISEQEETPRERMGDYIESFWRDQPRP
VHKVLEICCGTGLMLGDLQRRGYQVSGLDRSAAMLEQARNRLGTGVELVRAELPEI
PLHAGFDAVISAANGLTYLPGTGFGETLAAVARLLPPGGTFVFDLYGHGFFERFYDS
AEPRVLAVELEDVSYIWTFTAPPSRAHFDVVHSQFLRTPDAEAGTYTRTRELHRFHE
HTHTSVRRLAAEAGFSSAEVHDNWTSRPSTPESMYDTWTLTRGVLEHHHHHH

ATGACCGTTGATGTTCCGGTTAGCTCGGACCCGTATGCAAATCTGGCAGCAAGC
TATGATCGTCTGGTTGATTGGGTTATTAGCGAACAGGAAGAAACCCCGCGTGAG
CGTATGGGTGATTATATTGAATCGTTTTGGCGTGATCAGCCGAGGCCGGTTCATA
AAGTTCTGGAAATTTGTTGTGGTACCGGTCTGATGCTGGGTGATCTGCAGAGGC
GTGGATATCAGGTTAGCGGTCTGGATCGTAGCGCAGCAATGCTGGAACAGGCAA
GGAATCGTCTAGGTACCGGGGTTGAACTGGTTCGTGCAGAACTGCCGGAAATTC
CGCTGCATGCAGGATTTGATGCAGTTATTAGCGCAGCAAATGGACTGACCTATCT
GCCGGGAACCGGTTTTGGTGAAACCCTGGCAGCAGTTGCACGTCTGCTGCCGC
CCGGTGGTACCTTTGTTTTTGATCTGTATGGTCATGGATTTTTTGAGCGTTTTTAT
GATAGCGCAGAACCGCGTGTTCTGGCAGTTGAACTGGAAGATGTTAGCTATATTT
GGACCTTTACCGCTCCGCCGAGCCGTGCACATTTTGATGTTGTTCATAGCCAGTT
TCTGCGTACCCCGGATGCAGAAGCAGGTACCTATACCAGGACCCGTGAACTGCA
TCGTTTTCATGAACATACCCATACCAGCGTTCGTCGTCTGGCAGCAGAGGCAGG
ATTTAGCAGCGCAGAAGTTCATGATAATTGGACCAGCCGTCCGAGCACCCCGGA
AAGCATGTATGATACCTGGACCCTGACCCGTGGGGTTCTCGAGCACCACCACCA
CCACCACTGA

SeMT_9x mutant (mutations are highlighted in grey)

MGHMTVDVPVSSDPYANLAASYDRLADWAISEQEETPRERMGDYIESFWRDQPRP
VHKVLEICCGTGLMLGDLQRRGYQVSGLDRSAAMLEQARNRLGTGVELVRAELPEI
PLHAGFDAVISAAGGLNYLPGTQFGETLAAVARLLPPGGTFVFDLFGHGFFERFYDS
AEPRVLAVELEDVSYIWTFTAPPSRAHFDMVYSQFLRTPDAEAGTYTRTRELHRYHE
HTHTSVRRLAAEAGFSSAEVHDNWTSRPSTPESMYDTWTLTRGVLEHHHHHH

ATGACCGTTGATGTTCCGGTTAGCTCGGACCCGTATGCAAATCTGGCAGCAAGC
TATGATCGTCTGGCAGATTGGGCAATTAGCGAACAGGAAGAAACCCCGCGTGAG
CGTATGGGTGATTATATTGAATCGTTTTGGCGTGATCAGCCGAGGCCGGTTCATA
AAGTTCTGGAAATTTGTTGTGGTACCGGTCTGATGCTGGGTGATCTGCAGAGGC
GTGGATATCAGGTTAGCGGTCTGGATCGTAGCGCAGCAATGCTGGAACAGGCAA
GGAATCGTCTAGGTACCGGGGTTGAACTGGTTCGTGCAGAACTGCCGGAAATTC
CGCTGCATGCAGGATTTGATGCAGTTATTAGCGCAGCAGGTGGACTGAATTATCT
GCCGGGAACCCAGTTTGGTGAAACCCTGGCAGCAGTTGCACGTCTGCTGCCGC
CCGGTGGTACCTTTGTTTTTGATCTGTTTGGTCATGGATTTTTTGAGCGTTTTTAT
GATAGCGCAGAACCGCGTGTTCTGGCAGTTGAACTGGAAGATGTTAGCTATATTT
GGACCTTTACCGCTCCGCCGAGCCGTGCACATGTTGATATGGTTCATAGCCAGTT
TCTGCGTACCCCGGATGCAGAAGCAGGTACCTATACCAGGACCCGTGAACTGCA
TCGTTATCATGAACATACCCATACCAGCGTTCGTCGTCTGGCAGCAGAGGCAGG
ATTTAGCAGCGCAGAAGTTCATGATAATTGGACCAGCCGTCCGAGCACCCCGGA
AAGCATGTATGATACCTGGACCCTGACCCGTGGGGTTCTCGAGCACCACCACCA
CCACCACTGA

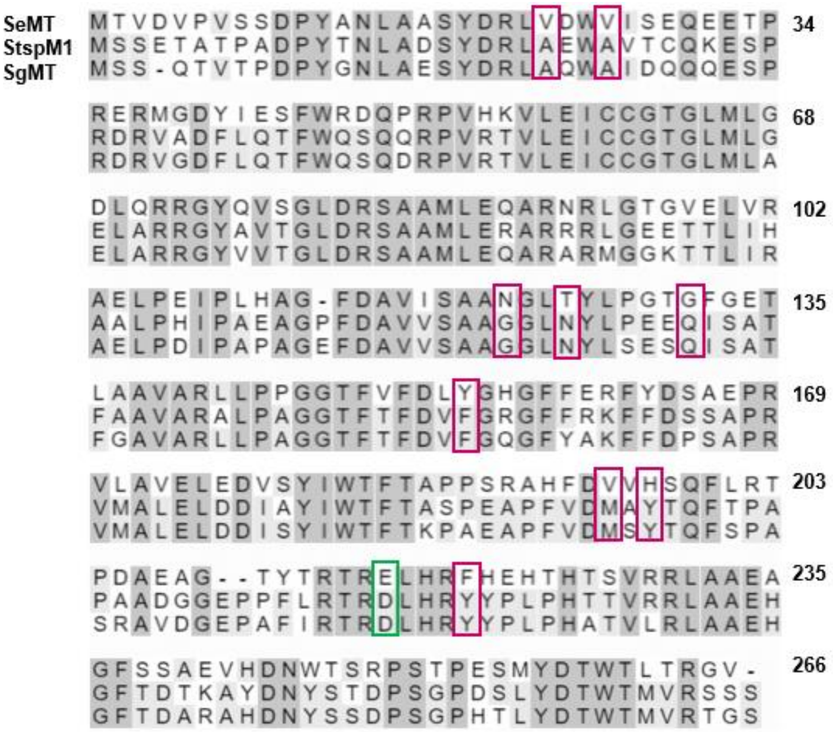

Figure S1: Sequences of SeMT, SgMT and StspM1. The nine mutation positions are highlighted in red, the 10th mutation position in green.

PCR conditions

Table S1: PCR components for the mutagenesis study.

Component	Volume (µL)
5X PrimeSTAR GXL Buffer	10
dNTP (2.5 mM)	4
Primer fw (10 µM)	1.5
Primer rev (10 µM)	1.5
Template vector (10 ng/µL)	0.7
PrimeSTAR polymerase	1
ddH_2O	31.3
TOTAL	50

Table S2: PCR conditions for the mutagenesis study.

Step	Temperature (°C)	Time	Cycles
1	98	30 s	
2	98	10 s	17
3	Touchdown	15 s	
4	68	3 min	
5	98	10 s	25
6	55	15 s	
7	68	3 min	
8	68	5 min	
9	4	storage	

SDS-Gels

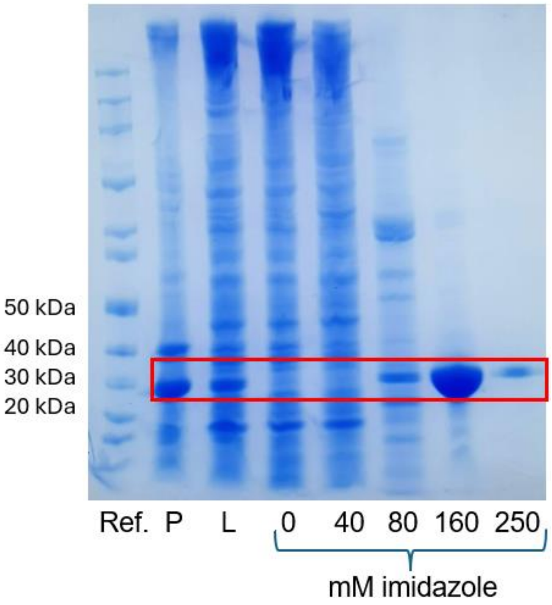

Figure S2: SDS-PAGE illustrating the purification of SeMT. As mass standard (Ref.) the Page Ruler unstained protein ladder (0.02–0.05 µg/µL) has been used. For the lysate (L), cell pellet after lysis (P) and flow-through (0 mM imidazole, ImH), 2 µL sample have been diluted in 10 µL water. The samples for the wash fractions (40 and 80 mM ImH), the elution fraction (160 mM ImH) and the purging fraction (250 mM ImH) were not diluted.

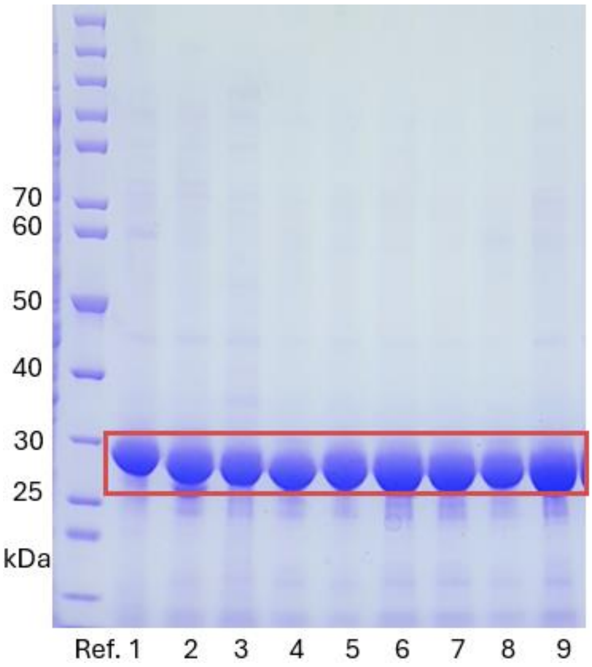

Figure S3: SDS-PAGE of purified SeMT variants: Wt (1), 10x mutant (2), 10x mutant with reverted mutations V23A+V26A (3), N122G+T125N (4), G131Q (5), Y154F (6), V195M+H197Y (7), E216D (8) or F220Y (9).

Crystallographic Data

Table S3: Data collection and refinement statistics. Values in parentheses refer to the highest-resolution shell.

Specimen	SeMT
PDB code	9ICZ
Data collection statistics	
Beamline	ESRF ID30B
Detector	EIGER2 Si 9M
Wavelength [Å]	0.873
Space group	$C\ 2\ 2\ 2_1$
Unit cell parameters	
a, b, c [Å]	153.0, 213.0, 48.2
α, β, γ [°]	90, 90, 90
Resolution range [Å]	62.12–1.58 (1.62–1.58)
No. reflections	106,524 (7,866)
Completeness [%]	98.4 (99.2)
Multiplicity	4.2 (4.3)
Mean I/σ(I)	11.4 (0.6)
$CC_{1/2}$ [%]	99.9 (30.6)
Refinement statistics	
No. of reflections used	106,441
R_{work}	0.173
R_{free}	0.193
RMS deviations from ideal	
Bonds [Å]	0.006
Angles [°]	0.760
Mean B [$Å^2$] (no. of atoms)	
Protein	38.7 (4948)
Ligands	51.0 (137)
Water	46.5 (668)
Ramachandran statistics [%]	
Favoured	97.7
Allowed	2.3
Outliers	0.0
Unusual rotamers [%]	0.8

Activity Data

Table S4: Methylation activity of SgMT, StspM1, and SeMT with seven different DKP substrates measured with the MTase-Glo Methyltransferase Assay at 45 °C. The enzyme concentration was set to 3 µM.

Substrate	*Consumed SAM [µM]*		
	SeMT	StspM1	SgMT
cWW	0.095	6.112	7.183
cWY	0	0.093	0.265
cWF	0	0.530	1.054
cWH	0	0	0
cWA	0	0	0
cWV	0	0	0
cWL	0	0.527	0.652

Table S5: Methylation activity of the 10x mutant of SeMT with seven different DKP substrates measured with the MTase-Glo Methyltransferase Assay at 25 °C. The enzyme concentration was set to 10 µM.

Substrate	*Consumed SAM [µM]*
cWW	8.052
cWY	0
cWF	0.861
cWH	0
cWA	0
cWV	0
cWL	0.135

Table S6: Activities of different SeMT variants measured with the Glo Assay using cWW as substrate. The reaction was carried out at 25°C for 15 min with an enzyme concentration of 10 µM. The 10x mutant contains all ten mutations from Figure 4a. The 1x mutant contains only the E216D mutation. The other variants are based on the 10x mutant, with the annotated mutations being reversed towards the wild type leading to 9x or 8x mutants. (1): V23A+V26A, (2): N122G+T125N, (3): G131Q, (4): Y154F, (5): V195M+V195Y, (6): E216D, (7): F220Y.

SeMT variant	*Consumed SAM [µM]*
wt	0.534
1x	1.081
10x	7.472
(1)	7.029
(2)	8.121
(3)	6.990
(4)	6.504
(5)	6.719
(6)	2.347
(7)	5.162

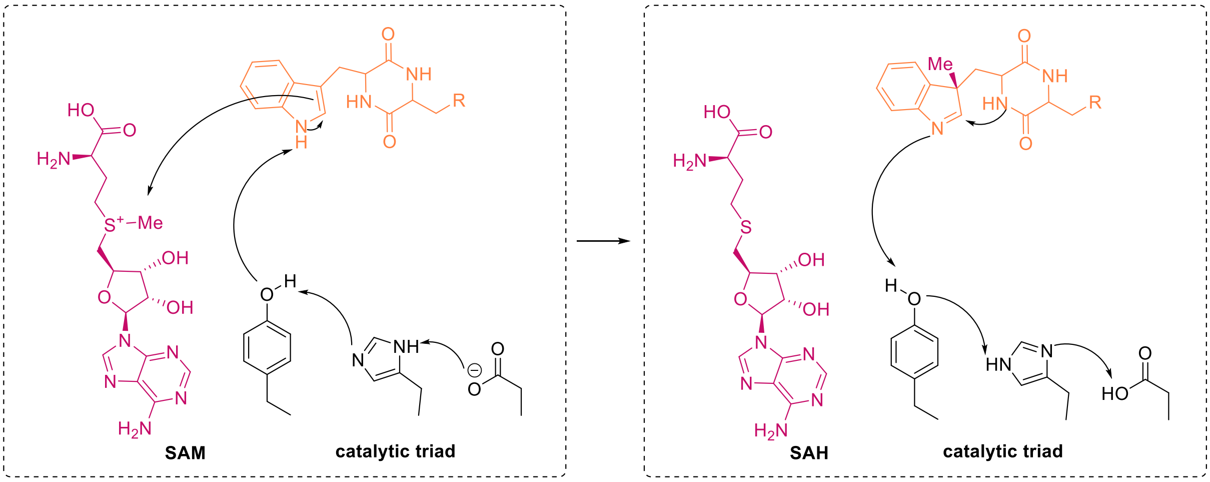

Figure S4: Mechanism of indole C3-MTases with a Trp-based DKP substrate (orange).

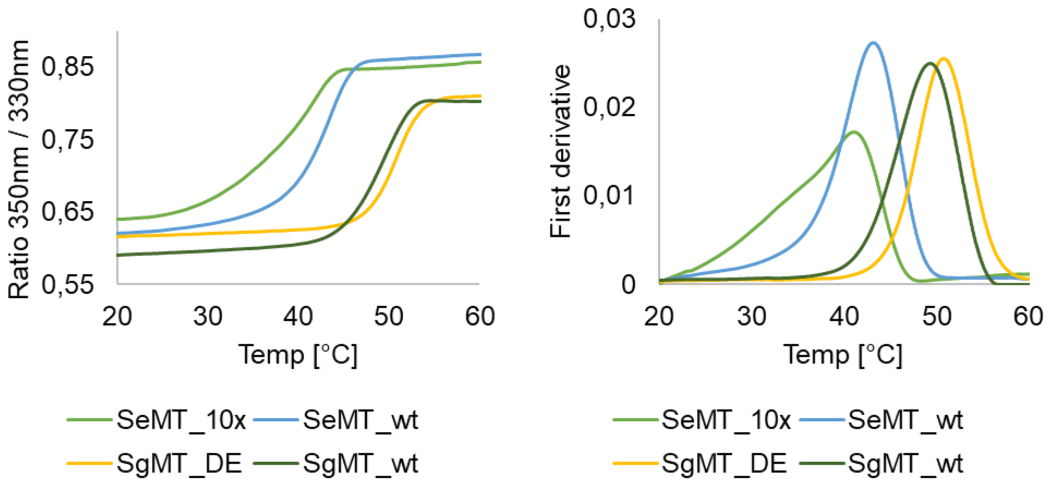

Figure S5: Thermal stability of SeMT (wild type and 10x mutant) and SgMT (wild type and D218E mutant), assessed via DSF. The ratio 350 nm / 330 nm was measured in triplicate (representative traces plotted) as a function of the incubation temperature (left), and the first derivative was calculated (right).

Table S7: Inflection points (peaks of first derivatives) from triplicate thermal unfolding (DSF) recordings of SeMT and SgMT variants (see Figure S5).

	Inflection points [°C]		
SeMT_10x	41.5	41.4	41.5
SeMT_wt	43.7	43.7	43.7
SgMT_DE	51.3	51.2	51.2
SgMT_wt	49.8	49.7	49.6
